# Mitochondrial Dysfunction in Aging, HIV, and Long COVID: Mechanisms and Therapeutic Opportunities

**DOI:** 10.3390/pathogens14101045

**Published:** 2025-10-16

**Authors:** María Victoria Delpino, Jorge Quarleri

**Affiliations:** Laboratorio de Inmunopatología Viral, Instituto de Investigaciones Biomédicas en Retrovirus y Sida (INBIRS), Universidad de Buenos Aires, Consejo de Investigaciones Científicas y Técnicas (CONICET), Buenos Aires 1121, Argentina

**Keywords:** mitochondria, HIV, SARS-CoV-2

## Abstract

We hypothesize that a unified mitochondrial perspective on aging, HIV, and long COVID reveals shared pathogenic mechanisms and specific therapeutic vulnerabilities that are overlooked when these conditions are treated independently. Mitochondrial dysfunction is increasingly recognized as a common factor driving aging, HIV, and long COVID. Shared mechanisms—including oxidative stress, impaired mitophagy and dynamics, mtDNA damage, and metabolic reprogramming—contribute to ongoing energy failure and chronic inflammation. Recent advancements highlight new therapeutic strategies such as mitochondrial transfer, transplantation, and genome-level correction of mtDNA variants, with early preclinical and clinical studies providing proof-of-concept. This review summarizes current evidence on mitochondrial changes across aging and post-viral syndromes, examines emerging organelle-based therapies, and discusses key challenges related to safety, durability, and translation.

## 1. Introduction

Mitochondria are central hubs for cellular metabolism, redox signaling, and innate immunity. Their dysfunction thus impacts more than just energy production, affecting tissue health and overall systemic well-being [1,2]. Viral infections often disrupt mitochondrial stability, either directly through interactions between viral proteins and organelles or indirectly via ongoing inflammation and oxidative stress. This damage can persist well after the virus is cleared, especially when mitochondrial DNA (mtDNA) is harmed, resulting in lasting bioenergetic problems and immune system imbalance [3,4].

We propose that a unified mitochondrial perspective across aging, HIV, and long COVID will reveal shared pathogenic mechanisms and specific therapeutic vulnerabilities that are amenable to organelle-targeted interventions.

This phenomenon is well illustrated in HIV and, more recently, in long COVID. Both conditions are associated with structural and functional mitochondrial alterations that resemble features of accelerated aging [5,6]. These include impaired respiratory chain activity, increased reactive oxygen species (ROS), altered metabolic programming, and defective quality-control mechanisms such as mitophagy [7,8]. Such processes contribute not only to chronic inflammation but also to the development of comorbidities typically associated with advanced age [9].

Although aging, HIV, and long COVID have each been separately linked to mitochondrial dysfunction; to our knowledge, a systematic comparison emphasizing how shared mitochondrial alterations inform organelle-based therapies is lacking. In this review we (1) compare mitochondrial changes across aging, HIV, and long COVID to identify convergent mechanisms; (2) dissect how these shared pathways contribute to bioenergetic failure and chronic inflammation; (3) evaluate emerging organelle-based therapeutic strategies; and (4) discuss key translational challenges—safety, durability, and feasibility—and propose research priorities to advance clinical translation.

By outlining this roadmap, we guide the reader through a cohesive argument that links mitochondrial pathogenesis to specific therapeutic solutions, highlighting vulnerabilities that are overlooked when these conditions are considered in isolation.

## 2. Mitochondrial Dynamics in Energy Production and Cellular Regulation

Mitochondria are multifunctional organelles whose roles extend well beyond ATP synthesis and whose dysfunction underlies a wide spectrum of human disease. In the matrix, tricarboxylic acid (TCA) enzymes produce reducing equivalents (NADH, FADH2) that deliver electrons to the inner-membrane electron transport chain (ETC, complexes I–IV), driving proton translocation and establishing the electrochemical gradient that powers ATP synthase (complex V) and supports protein import and organelle quality control [10,11,12,13,14,15].

Dysfunction of ETC components—particularly complexes I and III—raises electron leak and ROS production. Low, regulated ROS levels are signaling mediators that influence differentiation, immune responses, and adaptive stress resistance (mitohormesis). In contrast, excessive ROS causes oxidative damage, inflammasome activation, and pro-inflammatory signaling implicated in aging, HIV, and severe/long COVID [8,16,17,18,19]. Thus, the balance between bioenergetic competence and redox homeostasis is a central mechanistic node linking metabolic, infectious, and degenerative pathologies.

Beyond energy conversion, mitochondria supply precursors and cofactors for biosynthetic processes (pyrimidine and lipid synthesis), conduct fatty-acid β-oxidation, and assemble heme and iron–sulfur (Fe-S) clusters essential for respiration and DNA repair [20,21]. They also act as dynamic Ca^2+^ buffers that shape cytosolic and endoplasmic reticulum signaling, thereby influencing secretion, contractility, cell fate decisions, and programmed cell death [22]. Mitochondria determine cellular capacity to respond to stress, infection, and metabolic demand by integrating these biochemical and signaling roles.

**Figure 1:**
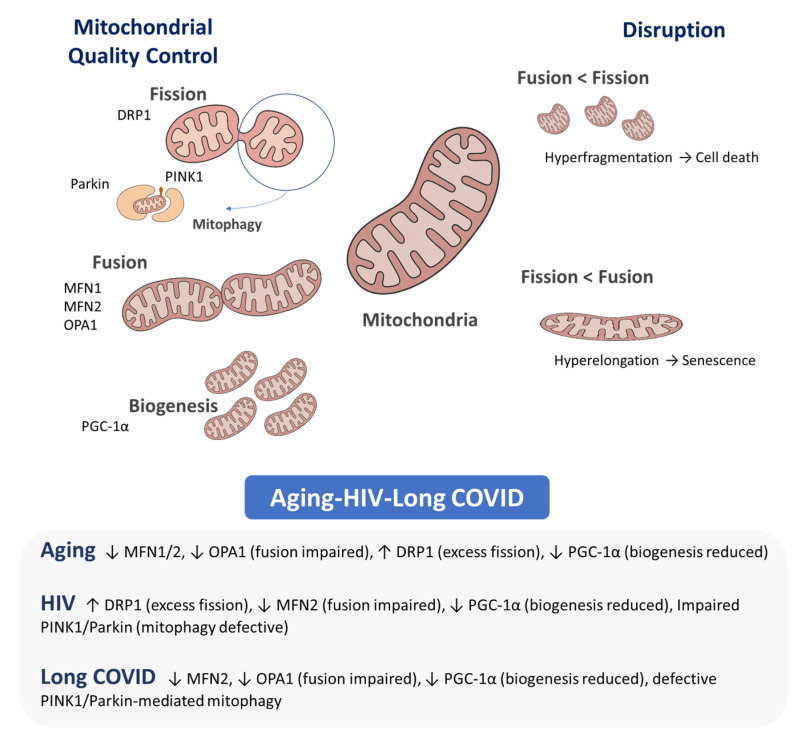
Mitochondrial dynamics. Mitochondria cycle through biogenesis, fusion, fission and mitophagy; balance among these processes preserves mitochondrial integrity and function. Key mediators—Fusion: MFN1/2 (outer-membrane fusion, network integrity), OPA1 (inner-membrane fusion, cristae maintenance); Fission: DRP1 (scission); Mitophagy: PINK1 (damage sensor), Parkin (E3 ligase); Biogenesis: PGC-1α (master coactivator of mitochondrial biogenesis). Alterations: Aging: ↓ MFN1/2, ↓ OPA1, ↑ DRP1, ↓ PGC-1α. HIV: ↑ DRP1, ↓ MFN2, ↓ PGC-1α, impaired PINK1/Parkin. Long COVID: ↓ MFN2, ↓ OPA1, ↓ PGC-1α, defective PINK1/Parkin.

Importantly for infectious disease, mitochondria are central platforms for innate immunity: mitochondrial antiviral signaling (MAVS), regulated fission–fusion dynamics, and mitophagy coordinate type I interferon responses, inflammasome activation, and cell survival programs. Viruses commonly target these interfaces to subvert host defenses—modulating fusion/fission, impairing mitophagy, degrading MAVS, or altering mitochondrial metabolism—to promote replication and persistence. Given this pathogen–host interplay, focused discussion of viral strategies that remodel mitochondrial form and function is essential (Figure 1).

Similarly, the therapeutic potential of mitochondrial transfer—through isolated mitochondria, organelle-bearing extracellular vesicles, or cell-mediated delivery—deserves recognition. Restoring organelle function provides a translational pathway to address bioenergetic failure in damaged tissues, but clinical application faces specific hurdles: ensuring long-lasting engraftment and functional recovery, preventing immune activation and harmful mtDNA heteroplasmy, standardizing isolation, storage, and potency testing, optimizing delivery methods (such as local injection, systemic mito-EVs, or cell carriers), and establishing meaningful endpoints and regulatory procedures.

## 3. Mitochondrial Aging

Aging has long been regarded as a degenerative process driven by the accumulation of damage at the cellular level, ultimately leading to tissue dysfunction and organismal decline. Among the numerous theories proposed to explain the mechanisms underlying aging, the mitochondrial free radical theory of aging (MFRTA) has dominated the field for several decades [23]. This theory posits that ROS, as byproducts of aerobic metabolism, are highly reactive molecules that induce oxidative damage to cellular macromolecules, including lipids, proteins, and DNA.

For years, the MFRTA has suggested that ROS-induced damage is the main cause of aging. However, recent studies have offered new insights into mitochondrial function in aging and challenged this long-held theory. It is clear that mitochondria have developed mechanisms to control physiological levels of oxidative stress, and that ROS alone cannot fully explain the aging process. For example, genetic mouse models have shown that somatic mtDNA mutations can cause progeroid phenotypes without a corresponding increase in oxidative stress [24,25,26]. This highlights the importance of mtDNA integrity in aging. Although the overall levels of mtDNA mutations in aging tissues stay relatively low, the clonal expansion of these mutations creates a mosaic pattern of ETC dysfunction, which contributes to functional decline in aging cells and tissues [27,28,29,30,31].

The connection between mitochondrial dysfunction, signaling pathways, and lifespan regulation has become clearer. Mitochondria are not just passive energy producers; they actively influence cellular signaling and metabolic processes that affect aging. For instance, caloric restriction (CR), a well-known way to extend lifespan, has been shown to improve mitochondrial function, indicating that better mitochondrial health may delay the onset of age-related diseases [32,33,34,35].

Despite these advances, many questions remain unanswered, including how mtDNA mutations accumulate during aging and the extent to which reducing these mutations can improve health and longevity. These challenges underscore the need for therapeutic strategies aimed at enhancing mitochondrial function as a means to combat age-related degeneration [36].

While the MFRTA has largely been refuted, the role of mitochondria in aging is undeniable [37]. Rather than viewing ROS as purely detrimental, it is crucial to recognize that these molecules also play signaling roles essential for adaptive stress responses and cellular homeostasis [38]. Understanding how mitochondrial dysfunction integrates with other hallmarks of aging will be key to developing interventions that promote healthy aging and potentially extend human lifespan.

## 4. Comparative Analysis of Mitochondrial Dysfunction in Aging, HIV, and COVID-19

Mitochondria play a key role as metabolic hubs, influencing not only the aging process but also the development of chronic viral diseases such as COVID-19 and HIV [7,39]. At first glance, aging and chronic viral infections may seem unrelated; however, they share important underlying mechanisms, including mitochondrial dysfunction, oxidative stress, metabolic disturbances, and immune system activation [40,41,42]. Importantly, these shared mechanisms also encompass impaired mitochondrial quality control (mitophagy) and disruptions of mitochondrial dynamics (fusion and fission), which together determine organelle integrity and cellular resilience.

These parallels highlight the intimate link between mitochondrial health, disease, and aging, while emphasizing the distinct ways in which these processes evolve. Nevertheless, these associations remain controversial in parts of the literature: aging, HIV and long COVID are biologically and temporally distinct conditions, and the causal link between viral infection and sustained mitochondrial dysfunction is not uniformly established across studies.

With age, mitochondrial function commonly declines because of accumulating mtDNA mutations, impaired activity of the ETC, and a diminished capacity for mitochondrial biogenesis. Together, these alterations lead to excessive production of ROS, which amplify oxidative damage to lipids, proteins, and nucleic acids, ultimately driving cellular dysfunction and tissue degeneration [43,44]. Chronic viral infections, including HIV and SARS-CoV-2, place considerable stress on mitochondria. These viruses hijack host mitochondrial functions to support their replication and to dampen antiviral immune responses. In doing so, they often generate oxidative stress and inflammation—processes that closely resemble the biological hallmarks of aging [45,46,47]. Viral perturbations commonly affect pathways that regulate mitophagy and mitochondrial dynamics, shifting the balance of fusion and fission and altering post-translational modification and turnover of the fusion/fission machinery (e.g., phosphorylation, ubiquitination, SUMOylation). These effects can impair PINK1–Parkin–dependent mitophagy and other quality-control pathways [48]. Specific viral factors have been implicated: SARS-CoV-2 ORF9b interacts with TOM70 and interferes with MAVS/TOM70-dependent antiviral signaling, modifying mitochondrial protein import and innate immune signaling [49].

HIV proteins such as Tat, Nef and Vrp induce mitochondrial membrane depolarization, disrupt respiration, and trigger mitochondrial damage or mitophagy in infected and bystander cells [50,51,52,53,54,55].

Both viruses can also suppress mitochondrial biogenesis by perturbing PGC-1α/NRF1/TFAM signaling, thereby limiting the generation of new mitochondria and impairing organelle turnover [8,56,57,58].

Collectively, these disruptions promote accumulation of dysfunctional mitochondria with mtDNA and respiratory-chain damage, excessive ROS production, bioenergetic collapse and amplified inflammatory signaling that are linked to the pathophysiology of HIV disease and the chronic sequelae observed in long COVID.

Besides the acute phase of SARS-CoV-2 infection, long COVID—a condition marked by ongoing symptoms months after the initial infection—has been linked to mitochondrial dysfunction [59,60,61,62,63]. New evidence indicates that aging mitochondria might play a key role in developing long COVID, especially in people with existing metabolic disorders or older age [64]. During long COVID, mitochondrial functions are disrupted, with less mitochondrial biogenesis, reduced respiratory chain activity, and increased ROS production [65]. These changes are similar to those seen in normal aging, where mitochondrial dysfunction is a sign of cell aging and inflammation. Constant mitochondrial stress in long COVID can increase systemic inflammation, resembling the “inflammaging” pattern observed with aging, leading to a cycle of immune activation and tissue damage [66,67].

A central shared hallmark of aging and chronic viral diseases is oxidative stress. In aging, ROS produced by the mitochondrial electron transport chain—especially at complexes I and III—oxidize cellular macromolecules, impair function, and trigger inflammation [68]. Similarly, in COVID-19, excessive ROS production has been implicated in the cytokine storm observed in severe cases [69,70,71,72,73]. Excessive ROS generation has likewise been implicated in the cytokine storm of severe COVID-19 [69,70,71,72,73] and in long COVID, persistent mitochondrial dysfunction prolongs this injury, contributing to fatigue, myalgia, and cognitive deficits that resemble mitochondrial disease phenotypes [6,65,74,75,76,77,78,79,80,81]. In HIV, chronic oxidative stress drives systemic inflammation and immunosenescence, further reinforcing parallels with aging [82,83,84,85,86].

Clinically, people living with HIV (PLWH)—even those on long-term suppressive antiretroviral therapy (ART)—show a higher prevalence and earlier onset of age-related comorbidities such as cardiovascular disease, metabolic syndrome, bone problems, and neurocognitive disorders compared to matched uninfected populations [87,88,89,90]. This faster accumulation of multiple conditions reflects chronic immune activation, residual viral reservoirs, lifestyle factors, and cumulative ART exposure. Mechanistically, mitochondrial disturbances in PLWH result from several interacting sources: direct effects of HIV proteins on mitochondrial dynamics and function, inflammation-driven oxidative stress, and drug-related mitochondrial toxicity [50,83,91,92,93,94]. Importantly, drug-induced mitochondrial toxicity—particularly from older nucleoside reverse transcriptase inhibitors (NRTIs)—is among the best-established mechanisms linking HIV treatment to mitochondrial dysfunction. Mechanistically, many NRTIs inhibit mitochondrial DNA polymerase γ (POLG), causing mtDNA depletion, impaired ETC assembly and activity, and consequent bioenergetic failure; classic examples include zidovudine (AZT) and stavudine (d4T), which were associated with pronounced mitochondrial side effects [95,96,97,98,99,100].

Although older nucleoside reverse transcriptase inhibitors (NRTIs) were strongly associated with mtDNA depletion, newer ART regimens—including INSTI-based combinations and updated NRTI backbones—have lessened visible mitochondrial toxicity; however, subtle mitochondrial impairments and metabolic side effects still appear in some treated individuals and likely add to long-term comorbidity risk [93,94]. Consequently, in PLWH it is critical to disentangle mitochondrial alterations driven by the virus itself from those that are treatment-related—and to consider that in many cohorts the cumulative exposure to older, more toxic ART compounds may confound comparisons with aging or with untreated viral infection.

Therefore, “aging with HIV” reflects the interaction of infection-driven mitochondrial stress with treatment- and host-related factors that speed up age-related diseases [82,83,84,85,86,93].

Metabolic disruption is another key intersection. Aged cells often shift from oxidative phosphorylation (OXPHOS) to glycolysis—a Warburg-like reprogramming that reduces energetic efficiency and promotes metabolic exhaustion [101]. Both HIV and SARS-CoV-2 similarly rewire host metabolism toward glycolysis, with consequences for immune exhaustion and impaired tissue repair [8,102,103,104,105,106,107]. In SARS-CoV-2, this metabolic shift supports rapid viral replication and impairs the immune response, while in HIV, the reprogramming of immune cell metabolism contributes to their functional exhaustion [108,109,110,111,112]. SARS-CoV-2 alters host folate and one-carbon metabolism post-transcriptionally, enabling de novo purine synthesis despite shutting down host translation. Infected cells show glucose and folate depletion, and viral replication is highly sensitive to folate metabolism inhibitors like methotrexate [108]. High-content screens in human airway organoids identify Hypoxia-Inducible Factor (HIF)-1α–glycolysis and fatty-acid biosynthesis as actionable vulnerabilities for blocking SARS-CoV-2 [108], and HIF-1α–driven metabolic reprogramming in monocytes/macrophages enhances glycolysis while impairing T cell responses and epithelial survival, positioning HIF-1α as a therapeutic target for COVID-19 [110]. Long COVID exacerbates these disruptions, since aged or dysfunctional mitochondria cannot meet recovery energy demands, worsening fatigue and repair deficits.

In HIV, CD8+ T cells show elevated oxygen consumption, reduced glycolytic capacity, and dysregulated mTOR signaling; impaired glycolysis contributes to T-cell exhaustion, whereas early infection or viral controllers display less metabolic derangement—suggesting metabolic interventions might restore CD8+ function [111,113,114,115]. Conversely, increased OXPHOS and glycolysis in CD4+ T cells heighten HIV-1 susceptibility, and partial glycolysis inhibition reduces infection, viability, and viral amplification. It reveals metabolic vulnerabilities that could be exploited to target HIV reservoirs mtDNA mutations provide an additional overlap [112]. Somatic mtDNA mutations accumulate with age and, through clonal expansion, generate mosaic ETC dysfunction across tissues, contributing to decline [116,117]. Chronic viral infections (HIV, SARS-CoV-2) further drive mtDNA damage via oxidative stress and inflammation [7,118], a burden that may be compounded by ART-related mitochondrial toxicity [93]. Although mtDNA mutations are relatively low in many aging tissues, their amplification in chronic viral contexts highlights convergent aging–viral mechanisms.

Inflammation unites these processes. Aging is marked by “inflammaging,” a persistent low-grade inflammatory state partly driven by mitochondrial dysfunction [119]; chronic inflammation is likewise central to COVID-19 and HIV. Hyperinflammatory acute SARS-CoV-2 responses can persist as long COVID, and chronic immune activation in HIV accelerates aging and promotes age-associated comorbidities [120,121,122].

A mechanistic synthesis links HIV-associated mitochondrial dysfunction to immunosenescence: impaired mitochondria increase ROS and release mitochondrial DAMPs (e.g., oxidized mtDNA) that activate innate sensors (cGAS–STING, inflammasomes), sustaining type I interferon and proinflammatory signaling [123,124,125]. This milieu expands senescent and exhausted T cell populations with diminished proliferative and effector capacity; senescent cells secrete a pro-inflammatory SASP that impairs tissue homeostasis and repair. Concurrent metabolic rewiring (impaired OXPHOS, altered NAD+/NADH balance, dysregulated mitophagy) further undermines immune resilience, linking mitochondrial stress to systemic comorbidity risk in PLWH [82,83,84,85,86,119]. Taken together, the evidence supports some common mechanistic themes (oxidative stress, metabolic reprogramming, chronic inflammation) but also underscores that the relative contribution of direct viral effects, host age-related decline, and drug toxicity varies markedly between conditions and cohorts.

In conclusion, although aging, HIV and COVID-19 converge on ROS overproduction, they differ in predominant etiology and affected pathways. In aging, ROS generation is mainly associated with dysfunction of the ETC (complexes I and III) due to accumulated mtDNA mutations and progressive declines in biogenesis and mitophagy. In HIV, mitochondrial dysfunction arises from a combination of direct viral protein effects, chronic inflammation and, in some cases, antiretroviral-associated toxicity; this introduces membrane depolarization–mediated damage in addition to oxidative stress. In SARS-CoV-2 and long COVID, beyond ETC damage, viral proteins (such as ORF9b) interfere with mitochondrial import and signaling (TOM70/MAVS), and defective mitochondrial quality control (mitophagy) promotes accumulation of dysfunctional organelles that continuously produce ROS. These etiological differences have clinical and therapeutic implications.

Mitochondria are a nexus connecting aging and chronic/post-infectious syndromes such as HIV and long COVID. These conditions share oxidative stress, metabolic reprogramming, and chronic inflammation, yet differ in timing, progression, and underlying triggers. Dissecting both common and distinct pathways offers routes to therapies that preserve mitochondrial health across this spectrum of disorders.

## 5. Mitochondrial Dynamics in Aging and Viral Infection

Mitochondrial shape and function are constantly remodeled through cycles of fission, fusion, selective autophagy (mitophagy), and intracellular transport. Together, these processes define the morphology, number, positioning, and overall quality of the organelles within the cell. Since mitochondria carry their own genome, they also rely on continuous repair, biogenesis, and protein turnover to maintain proper function [126].

Fission allows the separation of damaged mitochondrial regions, making it easier to eliminate dysfunctional fragments, while fusion enables the exchange of matrix and membrane components between organelles, helping to dilute damage and sustain respiratory efficiency. However, under intense stress, excessive fission can trigger apoptotic pathways. Mitophagy acts as a quality-control mechanism by selectively removing mitochondria that are beyond repair, thereby limiting the release of pro-apoptotic factors and reducing oxidative stress. In addition, transport along microtubules ensures that mitochondria are delivered to regions of the cell with high energy or Ca^2+^ demands, securing local ATP availability and maintaining homeostasis. The fine-tuned regulation of these mechanisms is therefore crucial not only for mitochondrial health but also for broader cell-fate decisions [127,128,129] (Figure 1).

Aging, chronic HIV infection and long COVID all converge on impaired mitochondrial quality control—mitophagy, biogenesis and mitochondrial dynamics—leading to accumulation of dysfunctional mitochondria, ROS overproduction and chronic inflammation [37,47,65,130,131,132,133]. In aging, ROS generation is largely attributable to progressive ETC dysfunction (notably complexes I and III) driven by accumulated mtDNA mutations and reduced proteostasis, changes that promote chronic oxidative damage and “inflammaging” [43,68,116,117]. Age-related declines in mitophagy reflect reduced expression and activity of PINK1 and Parkin, favoring retention of damaged organelles and increased ROS and inflammation [134,135,136,137,138,139,140,141], while diminished biogenesis via downregulation of PGC-1α/NRF1/TFAM limits replacement of dysfunctional mitochondria [57,142,143].

In chronic HIV, mitochondrial injury results from a combination of direct viral protein effects that perturb membrane potential and autophagic flux, persistent immune activation that amplifies oxidative stress, and, historically, antiretroviral-associated mitochondrial toxicity; these factors disrupt mitophagy, alter fusion–fission dynamics through post-translational modification of MFN/OPA1/DRP1, and blunt biogenesis, promoting bioenergetic collapse and accelerated comorbidity [46,50,51,52,53,83,91,92,93,94,144,145,146].

In long COVID, beyond acute ETC injury, viral interference with mitochondrial import/signaling (for example, ORF9b–TOM70/MAVS) and sustained inflammatory/metabolic reprogramming impair mitophagy (PINK1–Parkin and other routes) and dysregulate fusion–fission balance (reduced effective fusion; inadequate segregation/fission), leading to persistence of ROS-producing mitochondria and prolonged symptoms such as fatigue and exertional intolerance [8,49,53,59,60,61,62,63,64,65,144,145].

These distinctions—mtDNA accumulation and declining turnover in aging, viral-protein plus inflammation and drug effects in HIV, and defective import/quality-control with persistent metabolic stress in long COVID—have therapeutic implications (e.g., prioritizing mitophagy and biogenesis enhancement in aging and long COVID, while minimizing ART mitochondrial toxicity and targeting inflammation/mitoprotection in HIV).

Therefore, aging, HIV and long COVID each rewire mitochondrial metabolism (reduced pyruvate dehydrogenase (PDH)/pyruvate oxidation, greater reliance on glycolysis, NAD+/NADH shifts) but differ in proximate drivers (intrinsic aging-related mtDNA accumulation vs. viral proteins + ART vs. post-infectious persistent signaling). Integrating pyruvate-centric assays into comparative studies will clarify which interventions (PDH-centric, mitophagy/biogenesis, anti-inflammatory metabolic modulators) are most likely to restore mitochondrial health in each setting [147].

**Figure 2:**
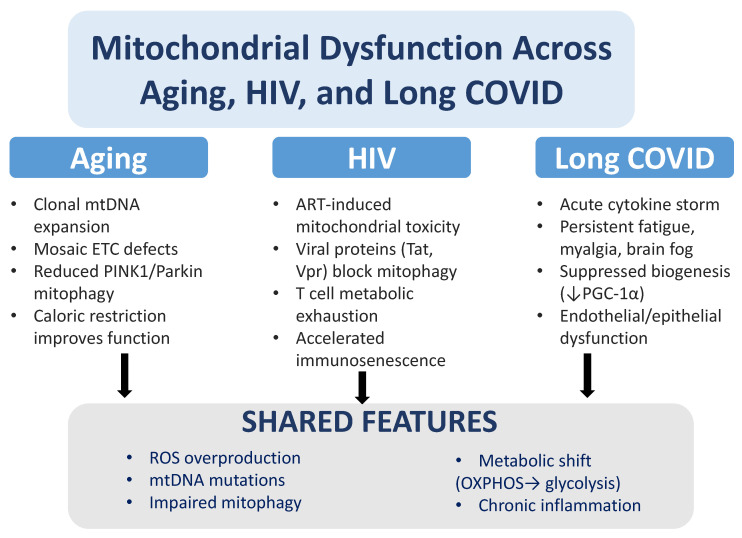
Mitochondrial dysfunction in aging, HIV, and long COVID. While aging, HIV infection, and long COVID affect mitochondria through distinct mechanisms, they converge on similar pathological outcomes. In aging, mitochondrial dysfunction is linked to clonal expansion of mtDNA mutations, mosaic defects in the electron transport chain (ETC), reduced PINK1/Parkin-dependent mitophagy, and improvements observed with caloric restriction. In HIV, antiretroviral therapy (ART) can cause mitochondrial toxicity, and viral proteins such as Tat and Vpr suppress mitophagy, driving T cell metabolic exhaustion and accelerating immunosenescence. In long COVID, cytokine storms, persistent fatigue, muscle pain, and brain fog are associated with reduced mitochondrial biogenesis (↓ PGC-1α) and endothelial/epithelial dysfunction. Across all three conditions, common features include excess reactive oxygen species (ROS) production, mtDNA mutations, impaired mitophagy, metabolic reprogramming from oxidative phosphorylation (OXPHOS) to glycolysis, and chronic inflammation.

Overall, the convergence of impaired mitophagy, mitochondrial biogenesis, and dynamics in aging, HIV, and long COVID underscores their role as critical drivers of cellular dysfunction, energy deficits, and chronic inflammation (Figure 1 and Figure 2, Table 1).

## 6. Mitochondrial Transplantation

Mitochondrial transplantation is a therapeutic procedure involving the isolation of functional mitochondria, which are then introduced into a living organism to integrate into target cells and restore or enhance cellular function [148].

An early groundbreaking study exploring the therapeutic applications of isolated mitochondria centered on transplanting healthy cardiac mitochondria into the rabbits’ hearts subjected to ischemia followed by reperfusion [149]. This pioneering approach demonstrated a notable reduction in ischemia–reperfusion injury and significantly improved heart function. Encouragingly, similar outcomes were later observed in studies involving pigs, further supporting the intervention’s potential [150]. Building on these findings, clinical trials explored the technique in pediatric patients requiring extracorporeal membrane oxygenation. By isolating skeletal muscle mitochondria from the patients and transplanting them back autologously, researchers accelerate recovery, allowing most patients to be successfully weaned off extracorporeal membrane oxygenation in a shorter timeframe [151,152]. More recently, mitochondrial transplantation promoted faster wound healing by reducing wound size, enhancing granulation tissue formation, and accelerating the process of epithelialization [153]. The effectiveness of platelet-derived mitochondrial transplantation in the treatment of ischemic heart disease was demonstrated in a randomized controlled clinical trial [154]. In addition, a mechanism underlying the effects of mitochondrial transfer between mesenchymal and endothelial cells is revealed when it is demonstrated that mesenchymal stromal cells (MSCs) use tunneling nanotubes to transfer mitochondria to endothelial cells (ECs) during cellular stress, and that preventing this transfer hinders EC engraftment [155]. Intercellular nanotube-mediated mitochondrial transfer was also used as a new immunotherapy approach, where healthy mitochondria are transferred from donor cells to T cells via nanotubes. This process boosts the T cells’ ability to produce energy, improving their function and reducing exhaustion. When applied in cancer therapy, these “supercharged” T cells become more effective at infiltrating and destroying tumors, leading to better tumor control and improved survival in patients [156].

Mitochondrial transplantation is classified into three primary types: autologous, heterologous (or allogeneic), and xenogenic transplants.

Autologous mitochondrial transplants involve isolating mitochondria from the same individual who will later receive them. This approach carries minimal risk since the mitochondria are entirely self-derived, ensuring compatibility and avoiding immune rejection [149,150,152].

In contrast, heterologous or allogeneic mitochondrial transplants rely on mitochondria isolated from another individual or a cell line. In some cases, mitochondria may be sourced from a biological mother or a relative within the same maternal lineage, as mtDNA is maternally inherited. Although clinical trials using heterologous mitochondria are still in preclinical stages [157]. However, in cases where donor mitochondria persist, the potential impact of mtDNA heteroplasmy on the recipient’s cells requires careful consideration. While extreme heteroplasmy has been linked to dysfunction [158], achieving such levels through transplantation appears unlikely based on current research [155]. Viral- and therapy-associated stresses accelerate mtDNA damage or alter heteroplasmy, which can produce focal respiratory chain defects and functional mosaicism in tissues; these lesions can persist and drive organ dysfunction [159].

Xenogenic mitochondrial transplantation, on the other hand, involves transferring mitochondria between species, such as from humans to mice. While this method serves as a valuable experimental model, its translational potential is limited due to the likelihood of immune reactions and incompatibility between mitochondrial and nuclear genomes.

A critical aspect of mitochondrial transplantation is its durability and mechanisms of action. For transplanted mitochondria to have a therapeutic effect, they must be taken up by recipient cells and integrated into their endogenous mitochondrial pool [160]. Studies suggest that metabolically compromised cells are more efficient at retaining and utilizing transplanted mitochondria [161]. Animal models of ischemia–reperfusion injury have demonstrated that donor mitochondria can persist in recipient tissues for up to 28 days [162]. However, recent findings reveal that transplanted mitochondria are often rapidly degraded by certain cell types, such as endothelial cells and macrophages [163,164]. While engraftment does occur in some instances, the therapeutic benefits of mitochondrial transplantation may also stem from indirect effects, such as the stimulation of mitophagy or mitochondrial biogenesis [155,165,166,167].

Not all mitochondrial transplants are identical, and their outcomes can vary significantly depending on multiple factors, including the source of mitochondria, the isolation process, and the route of administration [168]. Moreover, mitochondria can be engineered to target specific tissues [169]. These variations highlight the need for standardized protocols to improve the reproducibility of results.

To address this variability, it is recommended that mitochondrial transplantation studies provide detailed descriptions of the source material, isolation methods, mitochondrial size, and the composition of the product, whether it consists of free mitochondria or mitochondrial extracellular vesicles (Mito-EVs) [170,171].

In this context, MoDL, a deep learning-based software package, offers a transformative approach by bridging the gap between mitochondrial morphology and function [172]. Its deep learning algorithm facilitates precise segmentation of mitochondrial structures and accurate prediction of their functions using live-cell imaging data. Trained on a comprehensive dataset of super-resolution images annotated with functional biochemical data, MoDL empowers researchers to analyze heterogeneous mitochondria across various cell types. This software is particularly valuable in mitochondrial transplantation, where identifying healthy, functional mitochondria and predicting their therapeutic potential are critical. MoDL’s ability to generalize across diverse biological contexts makes it an indispensable resource for optimizing transplantation strategies, evaluating mitochondrial integration, and assessing therapeutic outcomes. By doing so, it drives progress in both fundamental research and clinical applications.

Moreover, any modifications made to the mitochondria after isolation should be transparently reported. Rather than aiming for absolute purity, researchers are encouraged to focus on enrichment, defined as the proportion of particles containing mitochondria, which can be quantified using techniques such as flow cytometry or immunoblotting [173,174,175].

As research in this field advances, a deeper understanding of the pharmacokinetics and pharmacodynamics of transplanted mitochondria will be essential. These unique biological therapies behave fundamentally differently from traditional small or large molecule treatments, necessitating tailored approaches for their characterization and clinical application. With these considerations, mitochondrial transplantation holds the potential to become a transformative therapeutic tool across a range of medical conditions.

## 7. Chronic Viral Infection and Mitochondrial Transplantation

Mitochondrial transplantation has emerged as an exciting and innovative therapeutic approach that offers a way to directly address cellular dysfunction by introducing healthy mitochondria into cells with impaired function. Over the past few years, this strategy has drawn considerable attention, particularly in conditions where mitochondrial health is profoundly compromised, as occurs in PLWH and long COVID [47,65,92,133]. Both conditions are characterized by shared underlying problems—chronic inflammation, oxidative stress, and disrupted energy metabolism—that make mitochondrial transplantation a potentially groundbreaking intervention.

However, the proposal to apply mitochondrial transplantation to chronic viral syndromes requires a much more explicit cell-biological justification, and it must be considered in the context of recent community recommendations [160]. The mechanistic rationale is currently incomplete: transplantation replaces organelles but does not necessarily correct upstream causes such as viral-driven mitophagy blockade or drug-induced mtDNA replication defects.

One of the most compelling advantages of mitochondrial transplantation is its ability to restore energy production within cells [176,177,178,179]. For PLWH or those stressed with the persistent effects of long COVID, mitochondrial dysfunction plays a central role in many of the symptoms they experience. Reduced ATP production in these conditions leads to lower energy availability in vital organs such as the brain, liver, and muscles, often resulting in debilitating fatigue and other systemic issues [63,78,79,92,180,181,182,183,184,185]. By transplanting healthy mitochondria, it might be possible to replenish energy stores and improve cellular function, offering hope for reversing these symptoms. Additionally, mitochondrial transplantation could help address the overproduction of ROS, a key driver of oxidative stress, which further damages cells and contributes to disease progression [186].

Membrane integrity and permeability are central, unresolved technical issues. Isolated donor mitochondria are highly sensitive to mechanical and oxidative damage during isolation and handling; loss of outer- or inner-membrane integrity or dissipation of the membrane potential (Δψm) will markedly reduce ATP-generating capacity and can provoke release of mitochondrial DAMPs (mtDNA, cardiolipin, formyl peptides) that trigger robust innate immune responses. The mitochondrial permeability transition pore (mPTP) and other membrane-permeabilizing events must be monitored because permeabilized organelles cannot provide the intended bioenergetic benefit and may instead increase inflammation or cell stress. These concerns are emphasized in the recent consensus statement, which recommends rigorous quality control of donor organelles (including Δψm and membrane intactness) before any translational application [160].

Importantly, mitochondrial transplantation could also influence the burden of pathogenic mtDNA variants that accumulate with age. By introducing functional mitochondria, the procedure may dilute cells’ load of mutated mtDNA and/or promote a shift in heteroplasmy towards wild-type genomes, thereby counteracting clonal expansion of deleterious mtDNA. However, this potential benefit must be balanced against the risk of creating new heteroplasmic mixtures; careful donor selection and longitudinal monitoring of mtDNA heteroplasmy will be required. Conversely, transplantation may create new heteroplasmic mixtures or introduce donor mtDNA variants; therefore, donor screening and longitudinal heteroplasmy monitoring (single-cell and bulk assays) are necessary safeguards.

Beyond energy restoration, mitochondrial transplantation may have profound effects on the immune system [187,188,189]. Chronic immune activation, a hallmark of HIV—even in individuals on effective antiretroviral therapy—plays a major role in disease progression [190]. Similarly, long COVID has been associated with persistent systemic inflammation, which affects various tissues and organs [191,192]. By improving mitochondrial health, mitochondrial transplantation could help modulate the immune system, dampening inflammation and restoring balance [193]. For example, CD4+ T cells, critical to immune defense but often dysfunctional in HIV, could benefit from the energy boost provided by healthier mitochondria, leading to better immune responses [194,195]. Likewise, in long COVID, improved mitochondrial function in endothelial and epithelial cells could reduce the widespread inflammation and vascular dysfunction that many patients experience [196,197].

Uptake routes and intracellular fate remain poorly defined and are decisive for efficacy. Key mechanistic gaps include: (i) how intact mitochondria cross the plasma membrane of different cell types (direct fusion, endocytosis, tunnelling nanotubes, or EV-mediated transfer); (ii) whether internalized mitochondria escape endolysosomal degradation; (iii) whether they retain Δψm and integrate (fuse) with host mitochondrial networks or are rapidly targeted for mitophagy; and (iv) the kinetics of persistence versus clearance in vivo. The consensus paper recommends minimal characterization assays (membrane potential, ROS generation, mtDNA release, endolysosomal co-localization) to address these unknowns prior to claiming therapeutic benefit.

In the context of HIV, where viral proteins can directly impair mitophagy and mitochondrial quality control, mitochondrial transplantation might be combined with strategies that restore mitophagy or inhibit the viral factors that block it. Thus, transplantation could both replenish functional organelles and work synergistically with targeted therapies to overcome virus-driven defects in mitochondrial turnover. However, unless upstream defects (e.g., POLG inhibition from prior NRTI exposure, chronic blocking of mitophagy by viral products) are corrected, transplanted mitochondria risk being subjected to the same dysfunctional quality-control pathways and cleared, limiting long-term benefit.

Mitochondrial transplantation could also offer targeted benefits for specific tissues. Neurocognitive issues, such as HIV-associated neurocognitive disorders and the brain fog reported by many long COVID patients, often stem from mitochondrial dysfunction in neurons [65,198]. The transplantation of healthy mitochondria into these cells could enhance their energy production, reduce inflammation in the brain, and potentially improve cognitive function. Moreover, mitochondrial transplantation might address cellular senescence—a process in which cells lose their ability to function properly but continue to contribute to inflammation [199,200]. Senescent cells are common in aging, HIV, and long COVID, and rejuvenating these cells through mitochondrial transplantation could restore tissue health and reduce chronic inflammation [201]. Yet CNS application faces major delivery barriers: the blood–brain barrier limits systemic access, and invasive routes (intrathecal or intracerebral) carry safety and scalability challenges. Carrier strategies (EVs/exosome-like particles, receptor-mediated shuttles, nasal delivery, transient BBB modulation) are being explored preclinically but require demonstration of safe biodistribution and lack of neuroinflammatory sequelae.

Targeting the central nervous system (CNS) presents significant practical challenges that are highly relevant to “brain fog” in long COVID and HIV. The blood–brain barrier limits access to systemically delivered mitochondria, so CNS-directed strategies (for example, intrathecal or intracerebral delivery, carrier systems such as extracellular vesicles or exosome-like particles, receptor-mediated transport, nasal administration, or transient BBB-permeabilizing approaches) are being explored preclinically. Each approach has distinct safety and scalability considerations, and successful CNS application will require rigorous assessment of delivery efficiency, off-target distribution, and neuroinflammatory risk.

Despite its promising potential, mitochondrial transplantation is not without challenges. One major concern is the risk of immune rejection [202,203]. Mitochondria from a donor, even if closely matched, may be perceived as foreign by the recipient’s immune system, leading to inflammation and negating the therapeutic benefits. Furthermore, the risk of introducing mitochondria with genetic mutations (in mitochondrial DNA) must be carefully managed, as this could worsen cellular function instead of improving it [204,205,206].

Another issue is the possibility of off-target effects. Transplanted mitochondria may integrate into unintended cells or tissues, potentially disrupting normal cellular processes [207]. This risk is particularly relevant for individuals with underlying inflammatory conditions, such as those seen in HIV and long COVID, where an overactive immune response could be further exacerbated. In HIV, an additional concern is the potential effect on viral reservoirs—latently infected cells that harbor the virus. ROS could act as promoters or reversers of latency, depending on the cell type. Therefore, ROS reverse HIV latency in macrophages but promote latency in T cells [208]. Improved mitochondrial function could theoretically activate these reservoirs, complicating disease management for patients already on antiretroviral therapy.

**Figure 3:**
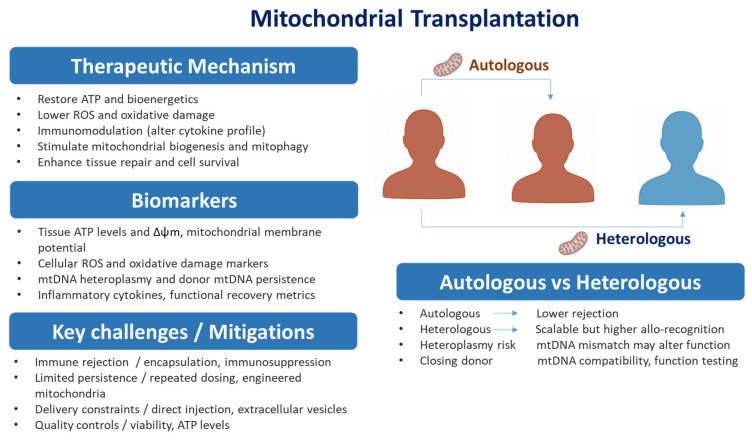
Mitochondrial Transplantation. Mitochondrial transplantation overview. Right: schematic of autologous (patient-derived—lower rejection, limited supply) versus heterologous (allogeneic—scalable, higher allo-recognition/heteroplasmy) approaches. Left: concise panels showing Therapeutic mechanisms, Biomarkers/options, and Key challenges/mitigations. Arrows link the routes to the panels; most strategies remain preclinical and require rigorous assessment of donor compatibility, safety, and persistence. Abbreviations: ROS, reactive oxygen species; mtDNA, mitochondrial DNA; Δψm, mitochondrial membrane potential.

The persistence of transplanted mitochondria also raises important questions. Early studies suggest that the introduced mitochondria can integrate into recipient cells and remain functional, but it is unclear how long these benefits last. In vivo studies have shown that donor mitochondria can persist in recipient tissues for up to 28 days [150,209,210]. These uncertainties must be addressed in future research. Furthermore, ethical and regulatory considerations—such as donor selection, mitochondrial compatibility, and the long-term safety of the procedure—require careful planning and oversight to ensure the technology is applied responsibly (Figure 3).

Recommended experimental and reporting roadmap (cell-biology-driven): (1) standardized quality control of donor mitochondria (Δψm, membrane intactness, absence of damaging mtDNA variants, redox state); (2) mechanistic uptake studies in primary human target cells (T cells, macrophages, endothelial cells, neurons) with assays for endolysosomal escape, fusion with host mitochondria, and mitophagy targeting; (3) immunogenicity testing in inflammatory milieu models (measure mtDNA release, cytokine induction, innate sensor activation); (4) heteroplasmy tracking and donor mtDNA fate over time at single-cell resolution; (5) biodistribution and persistence studies for each delivery modality (systemic, intrathecal, EV-assisted); and (6) adherence to the standardized nomenclature, minimal reporting criteria and safety endpoints recommended in the recent consensus [160]. These steps will help determine whether transplantation is mechanistically plausible and clinically safe for chronic viral conditions.

In summary, mitochondrial transplantation holds remarkable promise for addressing the mitochondrial dysfunction that underlies HIV and long COVID. By restoring cellular energy production, reducing oxidative stress, and modulating immune responses, this therapy could alleviate many of the systemic and tissue-specific problems that plague individuals with these conditions. Nevertheless, to address age-associated clonal mtDNA expansion, virus-mediated mitophagy blockade, and CNS-targeted symptoms such as brain fog, combined approaches (optimized donor selection, heteroplasmy monitoring, mitophagy-restoring agents, and targeted delivery systems) will likely be necessary. However, challenges such as immune rejection, off-target effects, and ethical concerns need to be resolved before this approach can be widely adopted. To fully harness the potential of mitochondrial transplantation, rigorous clinical trials and continued research into its mechanisms, safety, and long-term efficacy are essential. With these efforts, mitochondrial transplantation could emerge as a transformative therapy, offering hope to those living with the long-term effects of HIV and COVID-19.

## 8. Limitations and Future Directions for Mitochondrial Therapies in HIV, Aging, and Long COVID

Several important limitations shape our current understanding and point to priorities for future research. Much of the evidence on long COVID is still early and inconsistent, with wide variation in case definitions and cohort sizes. This makes it hard to draw firm causal conclusions or generalize findings. In people living with HIV (PLWH), the picture is even more complex: mitochondrial changes are influenced not only by the infection itself but also by aging, coexisting health conditions, and diverse histories of antiretroviral therapy (ART). These overlapping factors complicate efforts to separate drug-related mitochondrial injury from virus-driven effects.

Age-related clonal expansion of mtDNA mutations and heteroplasmy adds yet another layer of difficulty. While organelle transfer may dilute harmful mitochondrial genomes in some cells, it can also create new heteroplasmic mixtures with unknown long-term consequences.

On the technical side, several key issues remain unresolved. These include identifying the most suitable source of mitochondria, refining isolation methods, optimizing delivery routes, improving engraftment efficiency, managing immune recognition, limiting off-target uptake, and ensuring durable benefits. These challenges are particularly daunting when targeting the central nervous system, given the blood–brain barrier and the risks tied to intraparenchymal delivery.

Methodological inconsistencies also limit progress. Studies differ in their assays for viability and potency, their outcome measures, and even their reporting standards. Ethical and regulatory questions—such as how to select donors and how these therapies might affect viral reservoirs—remain unsettled as well.

Overcoming these challenges will require carefully designed studies. Only through such work can we determine whether mitochondrial therapies can safely and effectively correct the specific defects seen in aging, HIV, and long COVID, and guide their responsible translation into clinical practice.

## Figures and Tables

**Table 1:** Comparative summary of mitochondrial changes across Aging, Long COVID, and HIV. This table outlines representative proteins and viral factors, the status of key mitophagy pathways, fusion–fission dynamics, predominant sources of ROS, mtDNA integrity, and characteristic patterns of metabolic reprogramming across aging, long COVID, and HIV.

Feature	Aging	HIV (Chronic)	SARS-CoV-2/Long COVID
**Primary ROS Source**	ETC dysfunction—Complex I & III	ETC (I & III) + immune/inflammatory ROS; drug contribution	ETC (I & III) + damaged mitochondria accumulation
**Key Mechanisms**	mtDNA mutations; ↓ biogenesis; ↓ mitophagy	Viral proteins (Tat, Nef, Vpr); chronic inflammation; some ART toxicity	Viral interference (e.g., ORF9b–TOM70); impaired mitophagy; metabolic stress
**Mitophagy**	Mitophagy efficiency declines with age due to reduced PINK1/Parkin expression and activity, promoting accumulation of dysfunctional mitochondria, ↑ROS and inflammation.	Viral proteins (Vpr, Tat) disrupt autophagy/mitophagy flow, preventing clearance of damaged mitochondria; chronic inflammation and some ART regimens further impair mitophagy.	Persistent inflammation and metabolic reprogramming in acute/post-acute phases suppress mitophagy (PINK1/Parkin and other routes), promoting accumulation of dysfunctional mitochondria and prolonged symptoms.
**Fusion/Fission**	Reduced fusion (MFN1/2, OPA1) and excessive fission (DRP1) → fragmentation, loss of Δψm and energy deficits.	Altered fusion/fission balance driven by viral factors and inflammation; PTMs of fusion/fission machinery change network dynamics and can favor dysfunctional fragmentation.	Marked dysregulation: reduced fusion and inadequate fission, altered PTMs of MFN/OPA1/DRP1, impaired segregation and removal of damaged mitochondria.
**Biogenesis**	↓ PGC-1α/NRF1/TFAM, limiting generation of new mitochondria.	Often reduced due to chronic immune activation, oxidative stress and (in older regimens) drug-mediated inhibition of biogenesis pathways.	Reduced PGC-1α/NRF1/TFAM signaling in long COVID, limiting mitochondrial replication and repair.
**Metabolic Shift**	OXPHOS ↓ → ↑ glycolysis (partial)	Cell-type specific reprogramming	HIF-1α–driven glycolysis; persistent OXPHOS deficit
**Innate Activation (mtDAMPs)**	Chronic low-grade (inflammaging)	Persistent mtDAMP-driven inflammation	Ongoing mtDAMP release sustaining post-viral inflammation
**Clinical Signature**	Frailty, multimorbidity	Accelerated comorbidities; immunosenescence	Fatigue, exertional intolerance, cognitive symptoms
**Evidence Level**	High	High (variable)	Emerging

## Data Availability

No new data were created or analyzed in this study. Data sharing is not applicable to this article.

## Abbreviations

The following abbreviations are used in this manuscript:

mtDNA	Mitochondrial DNA
ROS	Reactive oxygen species
TCA	Tricarboxylic acid
ETC	Electron transport chain
MAVS	Mitochondrial antiviral signaling
MFRTA	Mitochondrial free radical theory of aging
CR	Caloric restriction
PLWH	People living with HIV
ART	Antiretroviral therapy
NRTIs	Nucleoside reverse transcriptase inhibitors
OXPHOS	oxidative phosphorylation
HIF	Hypoxia-Inducible Factor
DRP1	Dynamin-related protein 1
MSCs	Mesenchymal stromal cells
EC	Endothelial cells
